# Investigation of Carboxymethyl Cellulose (CMC) on Mechanical Properties of Cold Water Fish Gelatin Biodegradable Edible Films

**DOI:** 10.3390/foods6060041

**Published:** 2017-05-27

**Authors:** Mahsa Tabari

**Affiliations:** Department of Food Science and Technology, Faculty of Agriculture, Lahijan Branch, Islamic Azad University, Lahijan, Iran; m_tabari@iau-tnb.ac.ir; Tel./Fax: +98-21-2241-4573

**Keywords:** cold water fish gelatin, carboxymethylcellulose, edible films, Biodegradation

## Abstract

The tendency to use biocompatible packages, such as biodegradable films, is growing since they contain natural materials, are recyclable and do not cause environmental pollution. In this research, cold water fish gelatin and carboxymethyl cellulose were combined for use in edible films. Due to its unique properties, gelatin is widely used in creating gel, and in restructuring, stabilizing, emulsifying, and forming foam and film in food industries. This research for the first time modified and improved the mechanical properties of cold water fish gelatin films in combination with carboxymethyl cellulose. Cold water fish gelatin films along with carboxymethyl cellulose with concentrations of 0%, 5%, 10%, 20% and 50% were prepared using the casting method. The mechanical properties were tested by the American National Standard Method. Studying the absorption isotherm of the resulting composite films specified that the humidity of single-layer water decreased (*p* < 0.05) and caused a reduction in the equilibrium moisture of these films. In the mechanical testing of the composite films, the tensile strength and Young’s modulus significantly increased and the elongation percent significantly decreased with the increase in the concentration of carboxymethyl cellulose. Considering the biodegradability of the films and the improvement of their mechanical properties by carboxymethyl cellulose, this kind of packaging can be used in different industries, especially the food industry, as an edible coating for packaging food and agricultural crops.

## 1. Introduction

Preparing and producing packaging materials with the aim of maintaining quality, increasing the durability life and preventing environmental pollution have been among the research concerns and subjects for researchers in the food industry in recent years [1]. Pollution caused by packaging materials which are produced from oil derivatives and problems resulting from different disinfection methods such as burying, burning and recycling have recently attracted the attention of researchers to develop suitable alternatives for this packaging material [2,3,4].

In food industries, edible films and coatings are used for maintaining quality and increasing food durability. Development of high-performance renewable materials made from non-biodegradable polymers that can be degraded in nature, for producing bio-based recyclable packaging films is one important factor for sustainable growth of the packaging industry [5,6].

Gelatin is a protein compound derived from collagen which contains 18 types of amino acids and is in fact a heavyweight (10 to 20 kilodaltons) and water-soluble animal molecule which is obtained by hydrolysis from collagen thermal. Pure gelatin does not naturally exist but it is obtained from the hydrolysis of bone and cartilage of pigs and cows and, in lower amounts, from birds and fish [7,8]. Gelatin contains 84% protein, 14% humidity and 2% ash. It is soluble in hot water, ethylic alcohol, chloroform, ether and volatile and nonvolatile oil. Owing to its unique properties, gelatin is widely used in creating gel, and in reconstructing, stabilizing, emulsifying and forming foam and film in food industries. Gelatin films are transparent and are formed after gel drying [9,10]. The weak structure of fish gelatin and its low boiling point along with the low content of proline and hydroxyproline amino acids, which are mainly responsible for gel formation in cold water fish gelatin, have caused it to have weak mechanical properties [7,9,10]. To improve the properties of edible films and coatings, biopolymers are directly mixed in. Bio-composites or biodegradable composite films are composed of two or more biopolymers. Polysaccharides used in the packaging industry include starch, cellulose and its derivatives [11]. Carboxymethyl cellulose, which is a derivative of cellulose, is water-soluble, can form flexible and strong films by itself and is nontoxic and cheap. It is widely applied in food production owing to its high solubility in cold and hot water, ability to induce viscosity in solution, ability to form films, viscosity property, etc. [6,11,12]. To increase the mechanical strength and flexibility, plasticizers which contain materials such as water, acetone, fatty acids, glycerol alcohols, etc., are used for preparing films. They change the three-dimensional structure of the films and improve their tensile property. In addition to their high efficiency, they should be also nontoxic and edible [1,4,5,7]. The goal of this research was to produce biodegradable films based on cold water fish gelatin and to improve its mechanical properties by combining it with carboxymethyl cellulose, which can be used in the food industry.

## 2. Materials and Methods

To construct films, cold water fish gelatin was purchased from Canadian Sigma Company and carboxymethyl cellulose was purchased from Qazvin Glucosan Company. Also, liquid sorbitol and glycerol were purchased from Traco Liang (Penan, Malaysia).

### 2.1. Film Preparation

Weighting 4 grams cold water fish gelatin and dissolved in 50 mL deionized water than placed inside a water bath in 55 °C until a uniform solution is formed. Gelatin was dissolved in deionized water at 60 °C for 1 h. Carboxymethyl cellulose was dissolved in deionized water at concentrations of 5%, 10%, 20% and 50%. Then, by adding 1 to 3 ratio of sorbitol-glycerol plasticizer (sorbitol:3/glycerol:1), they were mixed with each other on a hot plate at 90 °C for 1 h using casting method [8]. A proper volume of the solution making film was poured by a pipette on plates made of poly methyl methacrylate (with trade name of Plexiglass) with size of 16 × 16 and thickness of 2 mL and was dried within 24 h in laboratory conditions (temperature of 25 ± 2 °C and relative humidity of 50 ± 5 °C). Then, they were isolated from surface of the plates and maintained inside a desiccator. The thickness of each film was then measured using a hand-held micrometer (Mitutoyo, Tokyo, Japan), at the nearest ±0.01 mm and eight different locations. All the films (including the control) were prepared in triplicate.

### 2.2. Mechanical Properties

A test to evaluate deformation (stretching) at constant speed in a sample of standardized dimensions was used to measure the resultant force required to rupture the material. From the curves of force vs. displacement a number of parameters were determined [13].

- **Tensile Stress** (also called tensile strength), σ, expressed in MPa. This corresponds to the measured force (N) required rupturing the section of the specimen [13]:
(1) σ = F/A,
  where F is the force in Newton (N) and A is the area of the section of the test piece (thickness width in mm^2^).
- **Elongation** (also called strain), ε (unit less). This is the ratio of displacement to length of reference sample [13]:
(2) $$\varepsilon =\frac{\Delta l}{l_{0}}=\frac{l-l_{0}}{l_{0}},$$
  where l is displacement (mm) and l_0_ is reference length (mm). Elongation at break is reported as a percentage relative to comparison flexibility of films.
- **Young’s modulus E (MPa).** This parameter corresponds to the slope in the linear stress-strain curve for low elongations [13]:
(3) $$E=\frac{\sigma }{\varepsilon },$$

The mechanical properties at each break were characterized. The breaking stress and strain (σ, ε) were calculated for each sample. The test section did not vary significantly during the measurement. The shape of stress-strain curves can be used to define a particular behavior of the material as brittle (breaking in the elastic range) or ductile (failure in plastic).

The American Society for Testing and Materials (ASTM) D882-10 with some modification was used to determine mechanical properties at standard conditions [13]. Film strips were cut to 100 mm long and 20 mm wide and conditioned for 48 h in 23 °C and 53% relative humidity [14]. Texture analyzer (TA.XT2, Stable Micro System and Survey, Godalming, UK) equipped with Texture Exponent 32 software was used for measuring mechanical properties of the films. The initial grip separation and crosshead speed were 50 mm and 30 mm/min, respectively. Elongation and tensile strength at break were calculated from the deformation and force data recorded by the software. Eight replicates for every sample were evaluated.

### 2.3. Sorption Isotherm

The moisture sorption isotherm at 25 °C determined based on the method described by Bertuzzi et al. [15]. The fitted Guggenheim-Anderson-de Boer (GAB) equation was used to estimate the moisture content of each film specimen surface in the permeability study. Absorption tests at equilibrium were measured in triplicate for each relative humidity and reported as g absorbed water/g dry film. Experimental sorption data were fitted using GAB equation [14]. The GAB model is defined by:

(4) $$w=\frac{w_{m}CKa_{w}}{\left(1-Ka_{w}\right)\left(1-Ka_{w}+CKa_{w}\right)},$$

where *w_m_*, *K* and *C* are the GAB parameters, *w* moisture content (dry basis) and *a_w_* water activity. To evaluate accuracy of GAB model for experimental sorption isotherm data for the films the percentage of mean relative deviation modulus (E) was calculated. It is given by the following formula:

(5) $$E=\frac{100}{N}\sum_{i=1}^{N}\frac{\left|m_{i}-m_{pi}\right|}{m_{i}},$$

where *N* is the number of experimental data, *m_i_* and *m_pi_*, are the experimental predicted value respectively. The modulus (*E*) value below 10% indicates a good fit practical [15].

## 3. Results and Discussion

### 3.1. Mechanical Properties

**Figure 1:**
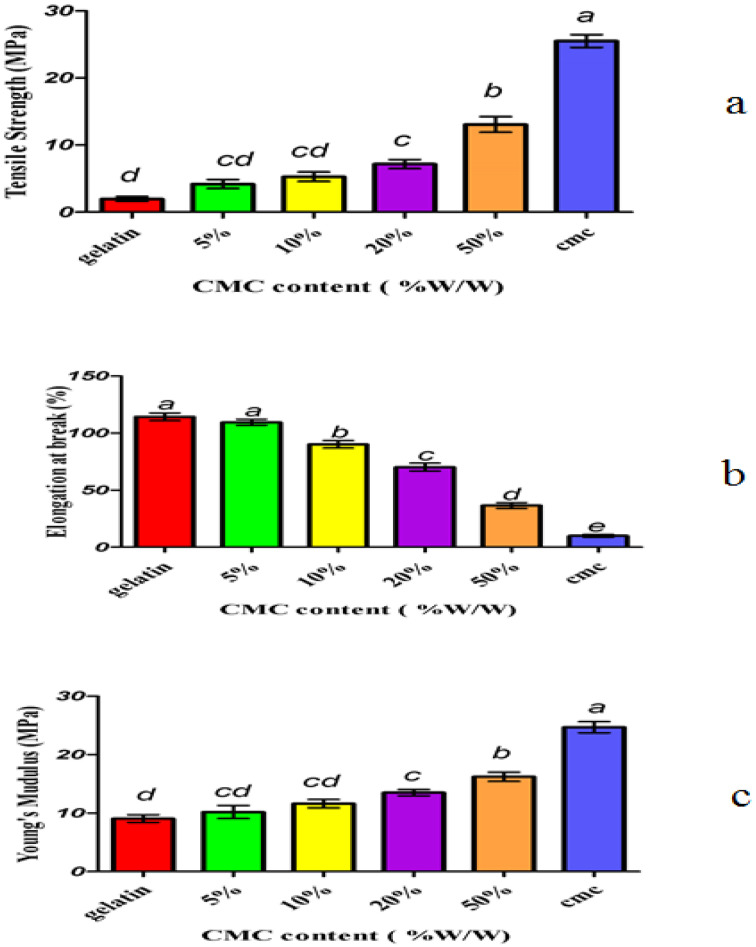
Percent tensile strength diagram (a), elongation diagram (b) and Young’s modulus diagram (c) with different concentrations of Carboxymethyl Cellulose (CMC) in cold water fish gelatin film. Different letters in each column indicate significant differences at the level of statistically 95% (*p* < 0.05).

Typically, the mechanical resistance of hydrocolloidal films has been studied according to three parameters: the tensile strength (TS), the Young’s modulus (Y) and the percent of elongation at break (E). The tensile strength, elongation properties, Young’s modulus are determined from stress-strain curves according to the norm of the American Society for Testing and Materials (ASTM) [13]. The results of the mechanical properties obtained from the texture analyzer are shown in Table 1 and Figure 1. The results demonstrated that the values of the tensile strength and Young’s modulus (stress-to-strain ratio in a linear area) in films containing carboxymethyl cellulose were higher than those of control films.

When the concentration of carboxymethyl cellulose was increased from 0% to 50% in the films, the hardness of the composite films increased from 1.9643 to 13.0677 MPa and the Young’s modulus increased from 1372.83 to 1842.87 kg/m^2^ (*p* < 0.05). With increasing the concentration of the carboxymethyl cellulose, elongation percent of the composite films was reduced from 114.335 to 36.507 (*p* < 0.05). Therefore, the improvement of the mechanical properties of the composite films was due to the surface interaction between the gelatin matrix and the fillers (biopolymers).

Gomez-Guillen et al. studied the effect of chitosan on cold water fish gelatin and concluded that long chains of polysaccharide macromolecules were bonded to the gelatin through cross-linking and thus increased the tensile strength of the composite films [10]. Chambi investigated the effect of gelatin and caseinate and concluded that polysaccharides could be bonded to a network of gelatin molecules and lead to the reinforcement of the structure and the mechanical properties of gelatin [16]. Lee, Shi and Lee examined the effect of gellan and gelatin in composite films and mentioned that the tensile stress of these films increased with the increase of gellan relative to gelatin, which occurred due to the intra-molecular reaction between polysaccharides and gelatin [17].

Some scientists reported that adding carboxymethyl cellulose to soy-isolated protein film improved the mechanical properties of these films compared with the control films and this increase was due to the reaction and bond of polymeric chains to proteins [18,19]. Qi reported that polysaccharides could bond to a network of gelatin molecules, leading to the reinforcement of the gelatin structure [5].

### 3.2. Absorption Isotherm

Studies have shown that an increase in the concentration of carboxymethyl cellulose decreases the amount of single-layer water of films (Mo), which indicates a reduction of their humidity content due to the hygrophilous property of carboxymethyl cellulose. Adding carboxymethyl cellulose improves the gelatin network so that gelatin can form hydrogenous bonds with the hydroxyl and carboxyl groups of carboxymethyl cellulose macromolecules, which leads to the reinforcement of the structure and the reduction of the diffusion of water molecules from the gelatin network in Table 2.

Similar results have been also obtained by other researchers who have observed that the addition of Dialdehyde Carboxymethyl Cellulose (DCMC) causes tensile strength and thermal stability to increase and elongation at break to decrease. Furthermore, the addition of DCMC can greatly decrease the water vapor permeability and the equilibrium swelling ratio. However, the addition of glycerol gives an increase of the elongation at break and the water vapor permeability and a decrease of the thermal stabilities and the equilibrium swelling ratio of the gelatin-DCMC films [20].

Increasing the CMC concentration in cold fish gelatin films could lead to enhanced humidity instead of reduced humidity. Ghanbarzadeh also produced films from corn starch with different amounts of citric acid and carboxymethyl cellulose [11]. An increase in the concentration of citric acid and carboxymethyl cellulose reduced the humidity absorption of the composite films [5,10,11].

### 3.3. Equilibrium Adsorption Diagrams

**Figure 2:**
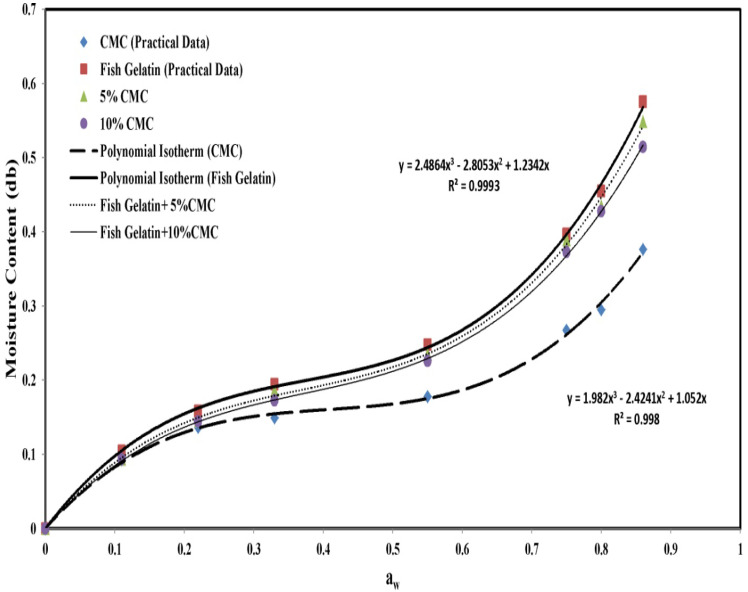
Moisture absorption of gelatin films as a function of CMC content.

The equilibrium moisture content was higher in fish gelatin films due to the fact that fish gelatin is more hydrophilic than CMC. Due to the presence of more hydroxyl groups in the molecule, fish gelatin interacted with water by hydrogen bonding. The results show that adding and changing the concentration of CMC is effective in reducing the equilibrium moisture absorption (Figure 2). These results were consistent with the results of Su et al. (2010) which showed that the addition of carboxymethyl cellulose to soy protein isolate decreased the equilibrium moisture absorption [19].

## 4. Conclusions

The studies conducted on composite films of cold water fish gelatin and carboxymethyl cellulose showed that:

1- An increase in the concentration of carboxymethyl cellulose reduced the equilibrium moisture and improved the mechanical properties of composite films compared with the control film. It also increased the tensile stress and film hardness and reduced the film elongation.
2- Since these films are made of biopolymer materials and one of the properties of biopolymers is their biodegradability, these films are biodegradable in the environment
3- One of the advantages of fish gelatin over other gelatins is its extraction from fish wastes. Composite films are also cost-effective.
4- Considering the obtained results, composite films showed better physical and mechanical properties than the control film and they can be used in the packaging industry.
5- In addition, cold water fish gelatin blended with carboxymethyl cellulose can be an alternative package material of some natural and synthetic products.

## Figures and Tables

**Table 1:** Results of mechanical properties of cold water fish gelatin films with added Carboxymethyl Cellulose (CMC).

Concentration	Tensile Stress	Elongation	Young’s Modulus
Control Fish Gelatin	1.9643 ± 0.024	114.335 ± 0.322	9.0625 ± 0.386
Control CMC (without Fish Gelatin)	25.5132 ± 0.020	9.9887 ± 0.054	24.7013 ± 0.386
Fish Gelatin with 5% CMC	4.1970 ± 0.015	109.505 ± 0.073	10.1975 ± 0.206
Fish Gelatin with 10% CMC	5.2875 ± 0.012	90.2236 ± 0.106	11.6250 ± 0.247
Fish Gelatin with 20% CMC	7.1542 ± 0.027	70.2231 ± 0.052	13.5125 ± 0.201
Fish Gelatin with 50% CMC	13.0677 ± 0.013	36.5075 ± 0.030	16.2275 ± 0.248

**Table 2:** GAB equation parameters (single-layer water of films (Mo), relative deviation modulus (E), *K* and *C* are the GAB parameters) for cold water fish gelatin films containing CMC at 20 °C. GAB, Guggenheim-Anderson-de Boer.

Concentration	M_0_	*C*	*K*	E (%)
CMC Control	0.101	147.492	0.845	7.91
Gelatin Control	0.140	29.545	0.889	7.13
5% CMC	0.139	29.588	0.882	7.99
10% CMC	0.137	29.796	0.873	6.49
20% CMC	0.129	67.460	0.869	6.54
50% CMC	0.114	73.291	0.853	6.78

## References

[1] Hu Z., Hong P., Liao M., Kong S., Huang N., Ou C., Li S. (2016). Preparation and characterization of chitosan—agarose composite films. Materials.

[2] Larotonda F.D.S., Matsui K.N., Sobral P.J.A., Laurindo J.B. (2005). Hygroscopicity and water vapor permeability of kraft paper impregnated with starch acetate. J. Food Eng..

[3] Choo K., Ching Y.C., Chuah C.H., Julai S., Liou N.S. (2016). Preparation and characterization of polyvinyl alcohol-chitosan composite films reinforced with cellulose nanofiber. Materials.

[4] Mariniello L., Giosafatto C.V.L., Moschetti G., Aponte M., Masi P., Sorrentino A., Porta R. (2007). Fennel waste-based films suitable for protecting cultivations. Biomacromolecules.

[5] Qi X.-M., Liu S.-Y., Chu F.-B., Pang S., Liang Y.-R., Guan Y., Peng F., Sun R.-C. (2016). Preparation and characterization of blended films from quaternized hemicelluloses and carboxymethyl cellulose. Materials.

[6] Rani M.S.A., Rudhziah S., Ahmad A., Mohamed N.S. (2014). Biopolymer electrolyte based on derivatives of cellulose from kenaf bast fiber. Polymers.

[7] Tripathi S., Mehrotra G.K., Dutta P.K. (2009). Physicochemical and bioactivity of cross-linked chitosan-PVA film for food packaging applications. Int. J. Biol. Macromol..

[8] Irwandi J., Faridayanti S., Mohamed E.S.M., Hamzah M.S., Torla H.H., Che Man Y.B. (2009). Extraction and characterization of gelatin from different marine fish species in Malaysia. Int. Food Res. J..

[9] Hamaguchp P.Y., Shiku Y., Tanaka M. (2003). Property improvement of fish water soluble protein films by dialdehyde starch (DAS) and/or sodium dodecyl sulfate (SDS) treatments. Pack. Sci. Tech..

[10] Gomez-Guillen M.C., Perez-Mateos M., Gomez-Estaca J., Lopez-Caballero E., Gimenez B., Montero P. (2009). Fish gelatin: A renewable material for developing active biodegradable films. Trends Food Sci. Technol..

[11] Ghanbarzadeh B., Musavi M., Oromiehie A.R., Razavi K., Razmi Rad E., Milani J. (2007). Effect of plasticizing sugars on water vapor permeability, surface energy and microstructure properties of zein films. LWT Food Sci. Technol..

[12] Masclaux C., Gouanvé F., Espuche E. (2010). Experimental and modelling studies of transport in starch nanocomposite films as affected by relative humidity. J. Membr. Sci..

[13] Annual Book of ASTM Standards (ASTM) (2010). Standard Test Method for Tensile Properties of Thin Plastic Sheeting D882-10.

[14] Van den Berg C., MacCarthy D. (1986). Water activity. Concentration and Drying of Foods.

[15] Bertuzzi M.A., Castro Vidaurre E.F., Armada M., Gottifredi J.C. (2007). Water vapor permeability of edible starch based films. J. Food Eng..

[16] Chambi H., Grosso C. (2006). Edible films produced with gelatin and casein cross-linked with transglutaminase. Food Res. Int..

[17] Lee K.Y., Shim J., Lee H.G. (2004). Mechanical properties of gellan and gelatin composite films. Carbohydr. Polym..

[18] Masclaux-Daubresse C., Daniel-Vedele F., Dechorgnat J., Chardon F., Gaufichon L., Suzuk A. (2010). Nitrogen uptake, assimilation and remobilization in plants: Challenges for sustainable and productive agriculture. Ann. Bot..

[19] Su J.F., Huang Z., Yuan X.Y., Wang X.Y., Li M. (2010). Structure and properties of carboxymethyl cellulose/soy protein isolate blend edible films crosslinked by Maillard reactions. Carbohydr. Polym..

[20] Mu C., Guo J., Li X., Lin W., Li D. (2012). Preparation and properties of dialdehyde carboxymethyl cellulose crosslinked gelatin edible films. Food Hydrocolloids.

